# Virologist Opinions: An Important Component for the Governance of the Convergence of Artificial Intelligence and Dual-Use Research of Concern

**DOI:** 10.1089/apb.2024.0060

**Published:** 2025-06-05

**Authors:** Matthew E. Walsh, Gigi Kwik Gronvall

**Affiliations:** ^1^ Department of Environmental Health & Engineering, Johns Hopkins Bloomberg School of Public Health, Baltimore, Maryland, USA.; ^2^ Johns Hopkins Center for Health Security, Baltimore, Maryland, USA.

**Keywords:** dual-use research of concern, artificial intelligence, governance, biosecurity, responsible development

## Abstract

**Background:**

The convergence of artificial intelligence (AI) and the life sciences has brought in silico research into policy conversations around dual-use research of concern and pathogens with enhanced pandemic potential research. This study considers the expert opinions of virologists on governance of AI and life sciences research.

**Methods:**

Semi-structured interviews with virologists were conducted and qualitatively analyzed to explore expert opinions about AI and virology. Interviewees were asked about the risks and benefits of AI, policy development considerations, and about evaluating the capability of AI tools in the field of virology.

**Results:**

Interviewed virologists generally expressed similar sentiments in responses to questions, including that benefits and risks of AI use in virology research are still to come, that policy and governance should be a process that includes virologist input, and that it is challenging to predict the capability of AI tools without experimental wet-lab validation.

**Discussion:**

Governance should be informed by expert opinions of practitioners, and it is important to consider how such opinions are incorporated. Expert opinions are valuable in understanding the impact of governance measures on beneficial research and development, and ensuring that governance measures are practicable and applicable. Virologists interviewed generally had similar opinions around AI and virology topics and often expressed an expectation that their opinions would develop over time.

**Conclusion:**

Given the uncertainty around the capability of AI technologies in the life sciences, it may be better to focus on developing frameworks for how governance measures will be developed, and to monitor developments, than to focus on specific interventions.

## Introduction

The impact of artificial intelligence (AI) technologies within the life sciences has recently become a focus of multiple U.S. government and advisory committee policies. A key concern is that these technologies could lower barriers to the development of biological weapons and democratize the ability to cause harm. The convergence of AI and the life sciences has thus brought in silico research into policy discussions surrounding dual-use research of concern (DURC) and pathogens with enhanced pandemic potential (PEPP) research.^1–3^ However, poorly designed policy-based risk mitigation measures could inadvertently limit the potential benefits of AI without meaningfully mitigating biological risk. To address this, we interviewed virologists to gather their opinions about the potential applications of AI in virology research and on proposed risk mitigation measures. This effort supports the development of rational, policy-based risk mitigation measures through publications like this one and informs our contributions as subject matter experts in interviews and conference and workshop discussions.

The United States released an updated policy guiding the oversight of DURC and PEPP research in May of 2024, which included limited discussion of in silico research.^4^ The new policy aims to unify federal governance of such research, expand the scope of oversight, and clearly delineate the roles and responsibilities of stakeholders involved in DURC and PEPP research. The new policy seeks to keep pace with scientific advancements and encourages, but does not require, institutional oversight of in silico research and the development of AI tools that can design novel pathogens. Set to take effect in May of 2025, is the result of a multi-year process marked by tension within the biosecurity, biosafety, and life science communities.

In February 2022, the National Science Advisory Board for Biosecurity (NSABB), a Department of Health and Human Services advisory committee, was tasked by the White House with evaluating the effectiveness of the two major U.S. policies governing DURC and research with PEPPs. Over the following year, the NSABB reviewed existing policies, consulted subject matter experts, and considered public comments. The NSABB published a report with their findings and recommendations in early 2023.^5^ Among its key findings and recommendations, the NSABB called for the unification of existing federal policies and expansion of the scope of research requiring federal review for potential DURC.

Many virologists, however, expressed concerns that the expanded scope recommended by the NSABB would encompass too much research where the benefits far outweigh the risks, including vaccine development, and potentially overburden those involved in the oversight process.^6^ Some virologists also argued that the NSABB’s recommendations were developed without adequate virology expertise.^7^ In apparent response to these concerns, the Office of Science and Technology Policy (OSTP), which was responsible for the development of the updated policy, issued a Request for Information (RFI) seeking feedback on the NSABB recommendations. While the NSABB did not recommended including in silico experiments within the updated policy’s scope, it did recommend continued assessment of their risks and benefits. As part of the RFI, the OSTP sought out opinions regarding whether in silico experimental approaches should require risk assessment and review. The updated policy, which incorporates many of the NSABB’s recommendations, goes a step further with regards to in silico experimentation. It encourages voluntary “institutional oversight of in silico research that could result in the development of potential dual-use computational models directly enabling the design of a PEPP or a novel biological agent or toxin.” Recognizing the ongoing development of AI technology, the update policy also commits to periodic review and potential updates at least every 4 years.

There are few examples of AI tools that produce outputs typically subject to oversight under traditional DURC/PEPP frameworks. For instance, researchers have developed AI tools capable of predicting SARS-CoV-2 viral escape mutants as accurately as some laboratory methods.^8^ However, there are significantly more AI tools developed outside the pathogen context that could theoretically be misused to engineer a pathogen. For example, tools intended to guide protein redesign to enhance desirable characteristics or tools that predict protein structures, could potentially be misapplied to engineer pathogen proteins instead of therapeutics or biotechnology-relevant enzymes.^9–11^ Governance of these tools will be challenging, as the concerning uses are often distinct from the intended applications. Additionally, it may be unclear how effectively these tools would function in misuse scenarios, further complicating efforts to assess and mitigate potential risks.

In addition to concerns about the misuse of biology-specific AI tools, there have been concerns about the potential misuse of general purpose chatbots (e.g., ChatGPT) by nefarious actors to aid in the development or production of biological weapons. These concerns are reflected in public policy. President Biden’s *Executive Order on the Safe, Secure, and Trustworthy Development and Use of Artificial Intelligence* describes AI tools as enabling the creation and proliferation of biological weapons.^12^ This Order is somewhat at odds with the way existing DURC/PEPP oversight frameworks operate. Current frameworks focus on overseeing how a technology (e.g., synthetic biology) is applied, rather than imposing limitations on the development or distribution of the technology itself. Provisions within the *National Security Memorandum on Artificial Intelligence* (AI NSM) look to address this discrepancy by requiring OSTP, National Security Council Staff, and the Office of Pandemic Preparedness and Response Policy to develop guidance around in silico biological research by April 2026.^13^

In the coming months and years, the governance of AI technologies within the life sciences will continue to mature. However, poorly designed policy-based risk mitigation measures risk inadvertently stifling the potential benefits of these technologies without meaningfully mitigating biological risk. Relying solely on the theoretical potential for misuse as a measure of risk may overestimate the likelihood of misuse and mischaracterize the negative impacts of a technology. Instead, incorporating an understanding of AI model performance and observed usage patterns may provide a more accurate assessment of the likelihood and risks of misuse.^14^ To inform the development of policy-based risk mitigation measures, we interviewed virologists to identify commonalties in their expert opinions surrounding the use of artificial intelligence in virology research and topics around policy-based risk mitigation measures.

## Materials and Methods

The Johns Hopkins Bloomberg School of Public Health Institutional Review Board Office determined that this research was not human subjects research (FWA #00000287).

### Interviews

Between May and July 2024, a series of interviews were conducted with 21 interviewees. A total of 75 potential interviewees were identified through authorship on peer-reviewed publications relating to virology policy and contacted for participation via email. Potential interviewees who were contacted held a PhD in the life sciences and typically were faculty at academic institutions. An additional nine potential participants were identified through snowball sampling and/or through review of websites of virology departments at major research universities that were not previously represented. In total, six potential participants declined to participate due to time constraints or self-determined lack of expertise in artificial intelligence. Three participants initially agreed to participate but did not respond to scheduling emails. The remaining 54 potential interviewees did not respond.

Researchers conducted semi-structured interviews via Zoom and followed an interview guide. The semi-structured interview format included predetermined questions and gave the interviewer the ability to ask additional questions based on the flow of the conversation. The interview guide with predetermined questions was developed based on three areas of active discussion around the incorporation of AI in the field of virology and the researchers’ personal experience and expertise related to the convergence of AI and the life sciences and associated policies. For each topic area, between 4 and 7 specific questions were included in the interview guide. Participants were introduced to the three topic areas at the beginning of the interview (Table 1), but were afforded the ability deviate from responding within the bounds of the pre-written questions. Interviewees were encouraged to respond based on whatever AI tool came to mind (e.g., ChatGPT or AlphaFold). All interviewees were provided an open-ended opportunity to share any additional thoughts at the end of the interview.

**Table 1.** Commonly asked questions, by category

Major category	Question
Risks and Benefits of artificial intelligence (AI) in Virology	• What are the benefits of AI in the life sciences broadly and virology specifically? • What are the risks of AI in the life sciences broadly and virology specifically? • Are there any experiments which should be off-limits from being conducted in silico?
Governance	• Who should be involved in developing policies relating to AI and virology? • What are the challenges to achieving effective policy? • How should the international community address these risks? Some foreign nations do not share the same values as the United States, how does this influence your thinking?
Risk Assessment and Evaluations	• What evidence would you need to see to convince you of the capability of an AI tool?

Interviews were typically around 45 minutes long and conducted on a not-for-attribution basis. Each interview was recorded with the interviewee’s consent and an automated transcript was generated using Zoom. The automated transcript was then reviewed against the recording and edited by a researcher to ensure the content of the transcript was accurate.

### Analysis

A qualitative approach was used to analyze the content of the interviews. One interview was conducted with an epidemiologist and infection preventionist but given their limited hands-on experience with virology research the interview excluded from analysis. Researchers reviewed the interview transcripts and identified seven areas of discussion for analysis based on their frequency. As the expertise of interviewees varied, not all questions in the interview guide were covered although each of the three major topic areas were. The areas for analysis typically aligned with specific questions from the interview guide. Each interviewee’s response was then coded to identify answers to the question as well as sentiments that were expressed throughout the conversations. The responses to each of these areas were then aggregated and described below.

## Results

Of the 20 interviews analyzed, 19 were with academic virologists (a list of interviewees is provided in the acknowledgements section). Interviewees studied multiple types of viruses, most commonly human influenza viruses, coronaviruses, and cytomegaloviruses, with one being the Director of a biosafety level (BSL)-3 laboratory. Interviewees came from 17 different U.S. academic institutions and two interviewees held a second terminal degree (one MD, one DVM).

### Risks and Benefits

When describing benefits of AI in virology and the life sciences, interviewees described virology-specific and broadly applicable benefits. These benefits corresponded to two categories of AI-tools: Large language model (LLM)-based chatbots and biology-specific AI tools.^15^ The virology-specific benefits typically represented enhanced or new capability. These included improved understanding of the relation between the structure and function of biomolecules, as well as broader advancements in understandings disease dynamics, spillover risk, and biological systems.

Interviewees also described generally applicable benefits of AI, which included significant resource savings, such as time and money. Multiple interviewees referenced AI’s ability to assist virologists in reformatting and analyzing large datasets, as well as extracting and summarizing information from text, such as peer-reviewed publications. Additionally, they highlighted AI tools’ capacity to help write code and to help non-native speakers communicate their scientific research more effectively.

When discussing risks, interviewees raised a range of concerns. Some specifically mentioned the potential for AI to contribute to DURC. Interviewees also cited the misuse of AI for data falsification and its potential to mislead users by providing incorrect or inaccurate information. These concerns stemmed partly from anecdotal experiences with AI systems producing incorrect outputs and partly from the lack of transparency with how some AI systems operate. This lack of transparency creates challenges in verifying or validating the output of black-box AI models during the peer-review process. Others expressed broader worries about the impact on public trust in science if AI-generated outputs were conveyed as scientifically proven when they were not. A few interviewees described concerns about de-skilling, noting that reliance on AI could lead to a diminished understanding of the underlying science and scientific method, and therefore reduced troubleshooting ability in the next generation of scientists.

All interviewees were asked if they believed there to be any experiments that should categorically *not* be conducted in silico. The responses to this question almost always indicated a belief that there should not be a defined set of experiments that are off-limits for conducting in silico. However, the rationale for these responses were varied. Some participants believed that meaningful risk was attributable to real-world experimentation and that, at the current time, any in silico or AI-based experiment would need to be accompanied by real-world lab work that is subject to various biosafety and biosecurity provisions. This would then raise the question of the value of doing experimentation in silico alone. Additionally, a few interviewees qualified their answers by saying that researchers working on certain pathogens should have no expectation of privacy while others felt that some experiments only made sense in a controlled environment where access was limited or restricted to a subset of individuals.

### Policy

The interviewees’ experiences and knowledge of ongoing policy discussions varied, with very few demonstrating a significant understanding of current policy conversations around the convergence of AI and the life sciences. As a result, conversations focused broadly on policy development rather than specific policy proposals. Overwhelmingly, interviewees emphasized the importance of involving subject matter experts in virology and artificial intelligence in the policy development process. Less frequently, they indicated national security experts, publishers, policymakers, and the public should be included. Interviewees typically favored a policy development process that incorporates subject matter expertise during the drafting phase, rather than drafting policies without such input and seeking feedback from experts afterward.

During a few conversations, interviewees highlighted various challenges, including the difficulty of developing policies for rapidly advancing technologies, the need for all stakeholders involved in policy development to have a solid technical understanding, and the politicized atmosphere surrounding science in the United States. While the importance of international coordination on scientific policy was often acknowledged, achieving consensus on a single, multilateral policy to govern international research was often viewed as an insurmountable challenge. However, establishing guidelines or reaching agreement as to what should be subject to a policy was seen as a more achievable goal.

### Risk Assessment and Evaluation

Many proposed policy-based risk mitigation measures rely on anticipating whether an AI model possesses specific capabilities that have been determined to contribute to biological risk. This interview section was initially intended to cover red teaming exercises and other risk analysis and evaluation methodologies that have been reported in the literature.^16–18^ However, most interviewees were unfamiliar with these evaluation approaches. Consequently, after the first few interviews, the scope was narrowed to focus on the evidence required to determine if an AI model could design a novel pathogen. While most participants acknowledged the difficulty of addressing the question due to the lack of a clear definition for a “novel pathogen”, the majority indicated that experimental wet-lab data would be necessary to substantiate such a claim. Although not all participants were asked this specific question, none suggested that in silico results alone would be sufficient to conclude that an AI model could design a novel pathogen.

### Cross-Cutting Themes and Sentiments

Interviewees often elaborated on their answers, providing rationale or anecdotal experiences to support their responses. In doing so, they often expressed sentiments that were shared across multiple interviews. Some of these commonalities are included in Table 2.

**Table 2.** Common themes and sentiments across interviews

Major category	Minor points
Cautious optimism about artificial intelligence (AI) in the life sciences	• In many cases, interviewees pushed back on the premise of a question. When this happened, it typically occurred in the context of the capability of AI tools and the interviewee expressing the opinion that AI tools would have a capability in the near future. • A view that tools would get better in the future • Personal anecdotes about AI not performing well where AI provided an incorrect or incomplete answer
Hesitation that AI tools could enable the design of “worse” pathogens	• Nature is the best at making pathogens worse, why do we think AI would be better? • The scientific community does not have the ability to routinely make pathogens worse, why do we think AI would be able to?
Trouble justifying why someone would use AI as part of a plan to misuse biology	• Biology can already be very deadly, there are good options to misuse biology • Biology can be unpredictable, so why not cause harm another way
Miscellaneous	• Concerns about politicizing science
	• Stating that they “should” be using AI more in their work
	• Questioning how regulation can be applied to someone working on a computer

## Discussion

Virologists generally share similar opinions about the governance of the convergence of AI and the life sciences. While they recognize AI as an emerging technology with significant promise, many expressed skepticism its current performance in contexts specific to their research. This skepticism extended to the near-term benefits and risks of biology-specific AI tools within the life sciences. When discussing frontier models, interviewees were even more doubtful about their ability to assist novices in crafting detailed experimental plans, aligning with prior evaluations of earlier versions of frontier models.^17^

Many interviewees expressed uncertainty in their opinions, noting an expectation that their perspectives will develop, and possibly change, over time and as they gain familiarity with AI and as the technology matures. This uncertainty emphasizes the importance of governance approaches that are adaptable and flexible, ensuring responsiveness to advances in and changing perceptions of AI technologies.

Multiple federal stakeholders are responsible for the governing the convergence of AI and the life sciences. The United States and the United Kingdom independently established AI Safety Institutes (AISIs) in 2023 to help develop safety and security guidelines for AI generally, and for the use of AI within the life sciences specifically. In the United States, the AI NSM designates the U.S. AISI as the lead entity on these efforts. Additionally, the AI NSM instructs the OSTP to develop specific “guidance to promote the benefits and mitigate the risks of in silico biological and chemical research”, in consultation with relevant stakeholders, of which the AISI is likely to be a key partner.

Involving subject matter experts in technical governance is crucial to ensuring policy measures are practical and to help understand and address potential implementation challenges. The opinions of virologists on some of the existing language in the updated DURC/PEPP policy may foreshadow definitional issues that OSTP will face when crafting a policy on in silico biological research. As stated in the updated DURC/PEPP policy, institutional review committees should oversee the development of AI tools that could be used to directly design novel pathogens or PEPP.

However, interviewees often took issue with a lack of specificity in the definition of a “novel pathogen.” When asked what information they would require to determine if a tool could design a novel pathogen, interviewees often first noted that this lack of specificity. For example, interviewees described how “novel” could refer to something that has never been seen before but is highly similar to something that has been (e.g., a SARS-CoV-2 variant) or could describe something with a never-before-seen function. The OSTP simultaneously released a companion document providing implementation guidance for the updated DURC/PEPP policy, which could serve as a venue for resolving definitional challenges.

When asked to set aside the definitional challenges, all interviewees who were asked about validating a model’s capability agreed that some level of wet-lab experimental data would be necessary to justify claims about an AI model’s ability to design novel pathogens. Some interviewees indicated that as examples of AI models with the ability to design novel pathogens are reported, they might require less wet-lab data. However, they also highlighted significant risks and limited, if any, benefits in generating what is theorized to be a novel pathogen purely to evaluate an AI tool’s capability, especially if the AI tool’s primary purpose is unrelated to pathogen design. Additionally, and as noted in the AI NSM, it will be important to ensure that such evaluations are not misconstrued as offensive biological weapons research.

The implementation guide for the updated DURC/PEPP policy provides a workable definition of “reasonably anticipated” as it relates to experimental outcomes in DURC/PEPP contexts. The OSTP could adopt a similar approach to guide judgements about whether an AI model possesses a particular capability. However, interviewed virologists described how the “black box” nature of some AI systems creates a significant challenge. Unlike DURC/PEPP experimental outcomes where there is a scientific basis for determining what is reasonably anticipated, the “black box” nature of AI systems makes it difficult to understand their operations and therefore predict their behavior and potential outcomes.

Another reason to involve subject matter experts in the development of governance is to ensure a clear understanding of the impact on the beneficial use cases, allowing for a well-informed weighing of risks and benefits. This sentiment was frequently expressed in interviews, often in the context of concerns about the politicization of science. Many interviewees indicated that these concerns stemmed expressed that this concern was derived from their experiences during the COVID-19 pandemic, where they perceived that politicized fear led to calls for overly broad restrictions on their work and that of others.

There are several frameworks available to guide the development of informed governance approaches that balance diverse stakeholder interests. For instance, some frameworks focus on addressing societal concerns, while others seek to balance innovation with security.^19^ The effective use of such frameworks depends on achieving broadly agreeable assessment of risks and benefits, which can themselves be evaluated using various methodologies.^14,20^

However, given that most virologists interviewed are not yet routinely using AI in their daily research, the risks and benefits of AI remain largely theoretical and carry significant uncertainty. This uncertainty makes it challenging to reach consensus on what the risks and benefits entail. Therefore, it is essential to devote time and effort to discuss and deliberate on how such assessments will be conducted. Doing so can increase the likelihood that the outcomes are broadly accepted and effectively inform governance decisions.

Opportunities to advance the technical and theoretical foundations of such evaluations and assessments are forthcoming. The NIH issued a new charge to the NSABB on November 21, 2024, to provide recommendations related to in silico research in life science settings. This charge directs the NSABB to recommend strategies to identify risks associated with in silico research that could contribute to the design of a PEPP and to develop methods for assessing risks and benefits against clear criteria. While this work would benefit from the expertise of virologists who routinely use AI models, the interviews conducted for this study suggest that such experts may be relatively few in number.

### Study Limitations

While this study identified commonalities and trends within the interviewees’ responses, it was not designed to explicitly elicit or evaluate areas of agreement or disagreement. Additionally, the interviews were primarily conducted with academic virologists based in the United States. The findings should not be generalized across virologists globally, to non-virologists in the life sciences, or to those working outside of academic contexts. Finally, this study was conducted shortly after the publication of the updated DURC/PEPP policy and during ongoing public policy debate about governance of AI and the life sciences. Given that this study spanned three months, participants interviewed later in the study period may have had more exposure to the updated DURC/PEPP policy, and therefore different opportunities to inform and develop their opinions compared to those interviewed earlier.

Additionally, this study specifically solicits and characterizes the opinions and views of one group of stakeholders in the governance of AI and the life sciences: virologists. As such, the findings represent only one piece of the broader conversation. Further research is needed to explore the perspectives of other stakeholder groups, such as biosafety professionals, non-virologist researchers within the life sciences, and AI model developers, to provide a more comprehensive understanding and to inform governance discussions.

## Conclusions

Incorporating subject matter expertise into the development of technology governance is essential to ensure that governance and risk mitigation measures are practical and do not unnecessarily hinder the beneficial applications of a technology. For the governance of the convergence of AI and the life sciences, virologists are one critical group of subject matter experts who must be consulted. However, there currently appears to be limited adoption of advanced, biology-specific AI tools in the routine research activities of virologists. Many virologists interviewed anticipated their opinions would change over time as their familiarity with the technology grows. Considering this, it may be more productive for those responsible for developing governance measures (e.g., the AISIs, NSABB) to prioritize establishing robust frameworks for assessing the risks and benefits of AI technologies in the life sciences rather than pursuing specific interventions at this stage. Establishing such frameworks can provide a basis for more informed and adaptive governance as both AI technologies and their applications in the life sciences mature.
